# Self-image and beauty perception among unsighted youngsters aged 12-36 years

**DOI:** 10.5935/0004-2749.2024-0156

**Published:** 2024-08-30

**Authors:** Gerson Gomes da Nóbrega Filho, Ketyanne Barros dos Santos, Julia Tannenbaum, Audina M. Berrocal, Camila Vieira Ventura

**Affiliations:** 1 Department of Ophthalmology, Fundação Altino Ventura, Recife, PE, Brazil; 2 Department of Visual Therapy, Altino Ventura Foundation, Recife, PE, Brazil; 3 Department of Psychology, Barnard College of Columbia University, New York, USA; 4 Department of Ophthalmology, Bascom Palmer Eye Institute, University of Miami, Miami, USA; 5 Department of Research, Fundação Altino Ventura, Recife, PE, Brazil; 6 Department of Ophthalmology, Hospital de Olhos de Pernambuco, Recife, PE, Brazil

Dear Editor,

We conducted a novel, analytical, cross-sectional study that focused on the perception of self-image and self-esteem among young unsighted and sighted individuals. The participants were asked to complete a comprehensive questionnaire that addressed self-image, self-esteem, eating habits, and social relationships. Body image satisfaction, sociocultural attitudes toward appea rance such as fat and muscle fat, and perception of pressure exerted by family members, peers, and social media were evaluated using specific scales^(1,2)^.

The study included unsighted (n=19) and sighted (n=19) youngsters. Most of the participants in both groups were female (unsighted group: n=12, 63.2%; sighted group: n=14, 73.7%). The mean age of the patients was 22.1 ± 7.2 years (range, 12.0-35.0) and 25.8 ± 4.8 years (range, 19.0-34.0) in the unsighted and sighted groups, respectively.

Our study revealed a significantly better self-image in the unsighted group than in the sighted group. Despite the lower educational and occupational levels (p=0.011 and p<0.001, respectively) and similar body mass index (p=0.064), the unsighted youngsters exhibited higher satisfaction with body appearance (p=0.006) and resistance to the influence of media standards of beauty (p=0.019) than sighted youngsters. This indicated that unsighted individuals had a higher self-esteem and were less susceptible to media pressure than sighted youngsters.

Physical beauty is a complex concept traditionally defined as a combination of qualities that evoke admiration or pleasure in the aesthetic sense. This may vary according to sex, social class, and cultural aspects^(3)^. However, due to globalization, the “standard of beauty” has become universal. Moreover, the ubiquitous exposure to social media amplifies the ideals of body image and promotes unattainable beauty standards^(4)^. Thus, youngsters face increased psychological pressure^(5)^. This pressure is especially marked in sighted individuals, who showed greater dissatisfaction with their body image than their unsighted peers. Our study findings demonstrated that sighted youngsters faced more psychological pressure due to the beauty standards established by the media. However, unsighted individuals are not as exposed to these ideals because of their physical limitation. Thus, their self-image and self-esteem develop in an environment that is not influenced by social media. This difference between the two groups raises awareness of the impact of social media on a youngster’s self-image and self-esteem.

The divergence in body image dissatisfaction between the two groups can be attributed to the lower exposure of unsighted individuals to visual media, reducing their vulnerability to society’s beauty norms. Furthermore, unsighted adolescents exhibited higher self-esteem and lower body dissatisfaction, despite the socioeconomic disparity between them and sighted individuals. These findings have significant implications for the health community and organizations dedicated to people with visual impairments. They highlight the intricate nature of beauty perception and self-image and the fact that factors beyond visual impairment can shape these aspects. Furthermore, our findings emphasize the need to consider these factors in initiatives aimed at promoting mental health and well-being among young individuals. Thus, our study findings are directly applicable and impactful and provide a foundation for more inclusive approaches to manage body image issues.

In conclusion, our study provides novel data regarding unsighted individuals’ perception of themselves. This perception results in a higher self-esteem than that observed in sighted individuals. Our study results highlight the remarkable resilience of unsighted individuals in the face of societal pressures for beauty. This resilience is a testament to the strength and adaptability of these individuals, a quality that should be celebrated and fostered. Furthermore, there is an urgent and ongoing need for approaches to protect adolescents from the influence of media pressure that may affect their self-image and self-esteem.
